# Impact of the COVID-19 pandemic on patients with chronic kidney disease: A narrative review

**DOI:** 10.1097/MD.0000000000029362

**Published:** 2022-06-17

**Authors:** Wanbing Huang, Bohou Li, Nan Jiang, Fengxia Zhang, Wei Shi, Li Zuo, Shuangxin Liu, Bin Tang

**Affiliations:** a Department of Nephrology, Blood Purification Center, Zhongshan City People's Hospital, Zhongshan, Guangdong, China; b Department of Nephrology, Guangdong Provincial People's Hospital, Guangdong Academy of Medical Sciences, Guangzhou, Guangdong, China; c Department of Nephrology, The People's Hospital of Gaozhou, Maoming, Guangdong, China; d Department of Nephrology, Peking University People's Hospital, Beijing, China.

**Keywords:** chronic kidney disease, coronavirus disease 2019, vaccination

## Abstract

Severe acute respiratory disease coronavirus 2 is currently causing the coronavirus disease 2019 (COVID-19) pandemic, placing extreme strain on the global health system. Vaccination is the main measure for preventing the COVID-19 epidemic, especially for high-risk groups including patients with chronic kidney disease (CKD). However, CKD patients receiving dialysis or kidney transplant may be characterized by decreased renal function and immune disorders, which may have uncertainties in their health. This overview aims to introduce the possible impact of the COVID-19 vaccine on kidney disease and its application in patients with CKD to provide evidence for the COVID-19 vaccine in patients with CKD. The data for this study were collected from PubMed, Cochrane Library, Embase, ClinicalTrials.gov, and the China Knowledge Resource Integrated Database (CNKI). The following keywords were used: “COVID-19”, “COVID-19 vaccine,” and “CKD”. The publication time of the papers was set from the establishment of the databases to September 2021. A total of 47 studies were included, and patients with CKD are a high-risk group for COVID-19 infection and severe illness. Vaccination is a powerful tool for preventing CKD patients from COVID-19. Because of possible side effects, the recurrence or deterioration of kidney disease may occur in CKD patients after vaccination. Although vaccination for patients with CKD remains a problem, with the advantages outweighing the disadvantages, stable CKD patients should complete a vaccination plan, and doctors should be aware of the recurrence or deterioration of kidney disease and close monitoring.

**Data access statement:**

Research data supporting this publication are available from the electronic databases of PubMed, Cochrane Library, Embase, ClinicalTrials.gov, and the China Knowledge Resource Integrated Database (CNKI).

## 1 Introduction

From the end of 2019 to the beginning of 2020, coronavirus disease 2019 (COVID-19), caused by the severe acute respiratory disease coronavirus 2 (SARS-CoV-2), has become a global pandemic. As of August 22, 2021, the number of confirmed cases worldwide exceeded 211 million, of which over 4.4 million have died.^[1]^ The new type of coronavirus is characterized by large genetic diversity, rapid mutation rate, high prevalence, and wide distribution. Vaccination is the main measure to prevent the COVID-19 epidemic. For patients with chronic kidney disease (CKD), including those suffering from the primary disease or receiving dialysis or kidney transplant, the use of immunosuppressive agents, or other therapeutic factors can reduce immunity against COVID-19. Therefore, CKD patients are a high-risk group for COVID-19 infection and severe disease, and vaccination seems to be given priority. However, considering that vaccination may result in vaccine complications, abnormal immune response, or recurrence of the primary disease, some countries still have certain restrictions on the vaccination of CKD patients. This article systematically describes the application and impact of the COVID-19 vaccination on CKD.

## 2 Methods

### 2.1 Research question

What are the impacts of COVID-19 on kidneys and patients with CKD?

What are the effects of the vaccine on COVID-19 and patients with CKD?

Whether the patients with CKD in some countries could be vaccinated against COVID-19?

### 2.2 Search strategy

The search was carried out in the electronic databases of PubMed, Cochrane Library, Embase, ClinicalTrials.gov, and China Knowledge Resource Integrated Database (CNKI). The following keywords were used: “COVID-19,” “COVID-19 vaccine,” and “CKD”. The Boolean operators “OR” and “AND” were used. Equivalent key words were used in Chinese databases. Due to the importance of the topic, no time or language limits were established for the search. The publication time of the papers was set from the establishment of the databases to September 2021. This narrative review does not require approval from an ethics committee review board or patients’ informed consent.

### 2.3 Study selection

This review was performed by collecting data on clinical trials, primary research, and reviews of electronic databases. Articles published in peer-reviewed scientific journals were also included in this study. For the selection of studies, first filtering was carried out on the title and abstract, identifying the presence of the keywords used in the search strategy. Finally, the full texts were reviewed to identify the studies.

## 3 Results

In total,46 studies were included. Thirty two research studies, 5 case reports, and 9 letters were used to build this review.

### 3.1 The impact of COVID-19 on kidneys and patients with CKD

Most of the initial clinical symptoms are common respiratory tract infections, such as fever, cough, and myalgia, or non-specific systemic symptoms, such as hyposmia, confusion, dizziness, vomiting, and diarrhea in the COVID-19 infected patients.^[2,3]^ COVID-19 infection is not limited to severe acute respiratory damage in the respiratory system but also affects multiple organs of the heart, kidneys, and liver, causing multifunctional disorders and even death.

Although kidney involvement is not the main feature of COVID-19 infection, the kidney is one of the target organs of COVID-19 infection, and the renal function of some patients with COVID-19 deteriorates rapidly.^[4–6]^ The main clinical manifestations of kidney involvement after COVID-19 are hematuria, proteinuria, and acute kidney injury (AKI), and some patients require renal replacement therapy.^[7,8]^ Among patients with COVID-19 treated in the intensive care unit (ICU), 20% to 30% of patients require renal replacement therapy.^[9,10]^ The main risk factors for patients with COVID-19 who develop AKI and require renal replacement therapy are male sex, diabetes, non-white people, obesity, CKD, hypertension, and advanced age. The severity of COVID-19 is also positively correlated with an increased risk of dialysis treatment. Severe COVID-19 infection can lead to pathophysiological mechanisms of AKI with the following factors: shock and renal insufficiency can lead to renal ischemia and acute tubular necrosis; COVID-19 can directly infect and replicate in kidney cells; COVID-19 infection causes high coagulation status, and thromboembolism may further affect renal perfusion, leading to transient renal ischemia and even renal infarction;^[11]^ and finally, the immune and complement function abnormalities caused by severe COVID-19 may also be related to kidney damage.

The underlying disease is an important factor affecting the rate, severity, and mortality of COVID-19.^[12]^ For patients with CKD, including dialysis and kidney transplant patients, these factors have an important impact on infection and prognosis. On March 12, 2020, we reported for the first time a case of a maintenance hemodialysis patient with COVID-19.^[13]^ The hemodialysis patient returned to Zhongshan from Wuhan in January 2020 and developed a cough. Corresponding examinations, such as imaging and other related examinations, confirmed the COVID-19 infection, and he was immediately transferred to a specialist hospital for treatment and continued hemodialysis in the isolation room. The remaining 42 dialysis patients who were likely to come into contact with COVID-19 patients due to dialysis in the same room were also transferred to a specialist hospital for 14 days of hemodialysis treatment in an isolation ward. After active treatment, the symptoms of the COVID-19 patient were relieved, and the nucleic acid test result was negative. The results of the COVID-19 nucleic acid testing of the remaining dialysis patients and medical staff were negative.

Most patients receiving central hemodialysis go to the dialysis center 2 to 3 times a week, and each dialysis lasted approximately 3 to 4 hours. In addition, they travel via public transportation. Therefore, hemodialysis patients must come into contact with a large number of other patients on the road and during the dialysis process, and the risk of contracting COVID-19 has increased significantly. Research data show that compared with the general population, patients undergoing hemodialysis in Flanders, France, and New York State, USA, are 5 to 16 times more likely to be diagnosed with COVID-19.^[14,15]^ Reports from France, the United Kingdom, Belgium, Italy, and the United States show that between 4 and 15 weeks of the first COVID-19 surge, 5% to 20% of hemodialysis patients were infected with COVID-19 during this period.^[16–19]^ As asymptomatic infections and testing restrictions, it is indicated that the infected population is even larger, serological studies from dialysis centers in London and New York show that the seroprevalence rate is 28% to 36%, and it increases to 70% in the dialysis department serving rehabilitation centers,^[20,21]^ and low-income and middle-income countries have reported similar data. Research from India showed that between March 2020 and December 2020, 9% of hemodialysis patients tested positive for COVID-19, which is 20 times higher than that of the general population.^[22]^ This shows that hemodialysis patients are susceptible to COVID-19 and a hemodialysis center is a high-risk place during the epidemic. It may be necessary to increase prevention efforts, establish comprehensive screening, isolate COVID-19 patients, and send them to designated hemodialysis centers, which can effectively prevent the spread ofCOVID-19 in hemodialysis centers.

Hemodialysis patients who are infected with COVID-19 are at least 30% to 130% more likely to be hospitalized for further treatment than those without CKD (after age adjustment), and the case fatality rate is approximately 16% to 32%.^[23–25]^ A retrospective study in China collected data on 7154 maintenance hemodialysis patients from 65 centers in Wuhan and demonstrated that 154 dialysis patients were diagnosed with COVID-19, with an infection rate of 2.2%, of which 22.9% were severely ill, suggesting that the prognosis of hemodialysis patients is poor after being infected with COVID-19.^[26]^ In another study, 187 (1.5%) of 12,501 dialysis patients were diagnosed with COVID-19, of which 117 (62.6%) were admitted to the hospital, with a fatality rate of 28.3%. Population-based analysis shows that, compared with the general population, hemodialysis patients have a higher relative risk of death after being infected with COVID-19.^[27]^

There was no significant difference in the possibility of COVID-19 infections in kidney transplant patients who received long-term immunosuppressive therapy compared to non-transplant patients with CKD, but the severity rate increased significantly. Banerjee et al reported 7 cases of kidney transplant patients infected with COVID-19, all of which showed respiratory symptoms and fever.^[28]^ Of the 7 patients, 6 needed to adjust the dose of immunosuppressive agents, 4 were transferred to the ICU, and 1 died on the 12th day after showing symptoms. In another study, 20 kidney transplant patients with COVID-19-induced pneumonia were characterized by a high risk of exacerbation and high mortality rate.^[29]^ In this study, 20 kidney transplant patients with a median transplant duration of 13 years were recruited. All patients discontinued immunosuppressants and started taking methylprednisolone. All patients experienced rapid clinical deterioration and increased oxygen demand; 6 patients developed AKI, 1 required hemodialysis, and the median time to death of the other 5 patients was 15 days after the onset of symptoms. Therefore, the condition of kidney transplant patients infected with COVID-19 can progress rapidly; some patients need to be transferred to the ICU for further treatment, and their mortality rate is relatively high.

It can be seen that among patients with CKD, including dialysis and kidney transplant patients, the COVID-19 infection rate is higher, resulting from impaired immune function. The hospitalization rate and risk of death after infection with COVID-19 were significantly higher than those in the general population.

### 3.2 The preventive effect of the vaccine on the COVID-19

To deal with the crisis of the COVID-19 pandemic, active research and development of a COVID-19 vaccine are key steps, and there is evidence that the COVID-19 vaccine can significantly reduce the infection rate. There are 4 main goals of the COVID-19 vaccine:

1. to reduce the deterioration of the condition and reduce the mortality rate after COVID-19 infection;
2. to protect the population at high risk of infection;
3. to prevent the spread of COVID-19; and
4. to maintain public life.^[30]^

Vaccination improves the body's ability to defend against infectious COVID-19 pathogens. Even when exposed, it can prevent infection or reduce the severity of the disease. Mass data shows that COVID-19 vaccines stimulate the body to produce sufficient immunity to maintain substantial efficacy against most mutant strains. The reason the COVID-19 vaccine can provide substantial protection to the body may be related to the extensive immune response they induce, which means that the mutation of the virus is unlikely to completely invalidate the vaccine. The Journal of the American Medical Association has published the results of phase III clinical trials of Sinopharm's COVID-19 inactivated vaccine. The results show that the 2 new inactivated vaccines of China Biologics produce high-titer antibodies and provide effective protection 14 days after the 2 injections. The protective effect was greater than 70%, and the positive conversion rate of neutralizing antibodies in the whole population was >99%. The protective efficacy of the WIV04 vaccine group (Wuhan Institute) was 72.8% and that of the HB02 vaccine group (Beijing Institute) was 78.1%. The safety of vaccines is good, and adverse reactions focus on pain at the injection site, which is mild, transient, and self-limiting.^[31]^

Except for the injection of the vaccine for the normal population, for patients with chronic diseases or corresponding treatment, the injection of the vaccine can reduce the hospitalization rate and mortality. In a retrospective study, 2005 COVID-19 positive patients over 18 years of age were included in the University of Florida healthcare system. The results showed that the hospitalization rate of patients who were not vaccinated against COVID-19 was 2.44 (95% CI, 1.68, 3.61) higher than those who had been vaccinated. The probability of being admitted to the ICU among inpatients was 3.29 (95% CI, 1.18, 13.77). These results were adjusted for age, race, sex, hypertension, diabetes, chronic obstructive pulmonary disease, obesity, coronary artery disease, and other influencing factors. This shows that vaccination has a potential protective effect in patients with moderate and severe COVID-19, and this protective effect is not affected by comorbidities.^[32]^ Another study evaluated the impact of 2 doses of the COVID-19 vaccine on reducing the incidence, hospitalization, and deaths due to COVID-19 in the United States. The results suggest that vaccination reduced the overall incidence rate from 9.0% in the never-vaccinated population to 4.6%. The highest relative reduction (54%–62%) was observed in people aged 65 years and older. Vaccination significantly reduced the adverse outcomes. Non-ICU hospitalizations, ICU hospitalizations, and deaths decreased by 63.5%, 65.6%, and 69.3%, respectively. It has been confirmed that even if the COVID-19 vaccine has limited protection against infection, vaccination can also have a significant impact on reducing the COVID-19 outbreak.^[33]^

### 3.3 Types of COVID-19 vaccine

Since the beginning of the COVID-19 epidemic, scientists worldwide have shared data and established cooperation to speed up the vaccine development process in a short period. According to information updated twice a week by the World Health Organization (WHO), as of August 27, 2021, 112 COVID-19 vaccines are in the clinical development stage, and 184 are in the preclinical development stage.^[34]^ In principle, the vaccination of infectious diseases is aimed at inducing the humoral or cellular immune response of the corresponding pathogen antigen so that the body's immune system can provide protection when it comes into contact with the pathogen. The main types of COVID-19 vaccines currently in clinical use include inactivated, adenovirus vector, and recombinant protein vaccines.^[35]^ As of June 3, 2021, as assessed by the WHO, the manufacturers that have reached the necessary safety and effectiveness standards for COVID-19 vaccines include AstraZeneca/Oxford Vaccine, Johnson & Johnson, Moderna, Pfizer/BioNTech, Sinopharm, and Sinovac.^[36]^ There are also live attenuated vaccines, protein subunit vaccines, virus-like particles, replicating and non-replicating virus vector vaccines, RNA vaccines, and DNA vaccines at the preclinical stage.

### 3.4 The possible damage of the COVID-19 vaccine to the kidneys

New types of COVID-19 vaccines have become the main means of responding to the COVID-19 pandemic and have played an important role in reducing medical and social public burdens. However, the side effects of COVID-19 vaccines must be noted. Recent studies have compared Pfizer/BioNTech mRNA and Moderna vaccines and pointed out that both vaccines can provide effective immunity against COVID-19 infection. However, both vaccines reported some related allergy symptoms, including pain, redness, or swelling at the vaccine injection site, fever, chills, fatigue, headache, muscle pain, nausea, vomiting, itching, joint pain, and anaphylactic shock.^[37]^ Several case reports have shown that patients with no previous history of kidney-related diseases have kidney damage after being vaccinated with the COVID-19 vaccine. A 52-year-old white man development anti-neutrophil cytoplasmic antibody (ANCA) glomerulonephritis 2 weeks after vaccination with the Moderna vaccine.^[38]^ Another elderly woman developed fever, anorexia, nausea, gross hematuria, and acute kidney injury 2 weeks after the same vaccine injection. Blood tests and renal biopsy pathology results showed anti-glomerular glomeruli with mesangial IgA deposition.^[39]^ It has also been reported that 2 men developed vasculitis after receiving 2 doses of the vaccine. However, many cases have reported that nephrotic syndrome occurred after the first dose of Pfizer mRNA vaccine, and pathological biopsy revealed minimal lesions.^[40,41]^

Currently, kidney disease may relapse after patients with CKD are vaccinated with the COVID-19 vaccine. Three patients with IgA nephropathy developed gross hematuria after vaccination with the mRNA-type COVID-19 vaccine, and their symptoms resolved spontaneously. Among them, 2 of 3 patients did not have anti-COVID-19 antibodies for serum detection.^[42]^ A 34-year-old woman had a history of nephrotic syndrome. The pathology was minimally changeable and was completely relieved by hormone therapy. After the Pfizer/BioNTech mRNA vaccine was injected, edema and proteinuria occurred. After increasing the dose of hormone therapy, proteinuria decreased, the second vaccination was administered (27 days after the first dose), and the disease recurred again, but it could be relieved by increasing the dose of hormone.^[43]^ A 66-year-old patient was diagnosed with IgG4-related nephropathy (IgG4-RD). After the related treatment, the symptoms, serum tests, and pathology indicated relief, and hormones were stopped. After the injection of the Pfizer/BioNTech mRNA vaccine, symptoms such as severe fatigue and myalgia occurred, and the level of anti-SSA-52 antibody increased, accompanied by acute kidney injury.^[44]^ Another 66-year-old female patient with previous primary membranous nephropathy was clinically in remission for 8 years and relapsed after being injected with the first dose of the inactivated vaccine. In addition, 2 patients with new-onset anti-PLA2R-positive membranous nephropathy were observed in patients with COVID-19, and it is speculated that COVID-19 may reduce the tolerance of PLA2R antigens.^[45]^ It is not clear whether the recurrence of these diseases is related to direct immune activation after vaccination or to chronic immune activation after an asymptomatic allergic reaction.

### 3.5 The prevention value of the COVID-19 vaccine in patients with CKD

Patients with end-stage renal disease (ESRD) undergoing maintenance dialysis or kidney transplantation have a higher risk of infection, hospitalization, and mortality than those in the general population. Therefore, the nephrology community recommends that ESRD patients, as a vulnerable group, prioritize vaccination. In the case of 160 chronic dialysis patients (127 hemodialysis and 33 peritoneal dialysis patients) inoculated with 2 doses of the V-2 BNT162b2mRNA vaccine for a 10-week observation period, the main findings were as follows:

1. the response rate to the vaccine was low;
2. anti-spike antibody levels were low (16 patients had negative antibody responses);
3. the hemodialysis group had a higher rate of COVID-19 infection after being vaccinated with COVID-19 (6 patients on hemodialysis were infected more than 7 days after completing 2 doses of vaccination).^[46]^

However, another study pointed out that vaccinated patients with weakened immune function may have a higher risk of contracting COVID-19 in its early stage.^[47]^ As of May 9, 2021, 25,088 Croatian citizens had been vaccinated with 2 doses of the COVID-19 vaccine, and 58 cases of symptomatic COVID-19 had appeared, including 8 kidney transplant patients. According to statistics, 0.02% of vaccinated individuals in the general population have COVID-19 infections and 4.8% have undergone kidney transplants.^[48]^

After receiving the COVID-19 vaccine, the antibody response rate was lower in dialysis and kidney transplant patients than that in the general population. Forbes et al conducted at least 148 days of follow-up in 122 hemodialysis patients diagnosed with COVID-19.^[49]^ The population remained antibody positive. The antibody gradient increased over time in 71% of patients and the use of immunosuppressive agents did not weaken the antibody response. This is an encouragement for the vaccination of CKD patients, including dialysis and kidney transplant patients. Speer et al^[50]^ collected data from 22 long-term hemodialysis patients and 46 healthy individuals from Heidelberg University Hospital between December 2020 and February 2021. After 2 doses of the BNT162b2 mRNA vaccine, the antibody response to the second dose of vaccine was low, with 82% of patients developing antibodies after the second dose, but the level was lower than that of healthy people.^[50]^ From February 3 to April 4, 2021, 3 dialysis centers in Barcelona, Spain, vaccinated 205 patients with mRNA-1273 (Moderna) or BNT162b2 (Pfizer/BioNTech) Covid-19, and followed up until April 2021 23rd. Of the 175 vaccinated patients who were seronegative at baseline, 97.7% had an immune response (humoral, cellular, or both) and 95.4% had seroconversion, where age and immunosuppressive therapy were associated with low antibody levels.^[51]^ Longlune et al tested the immune response of dialysis patients to a vaccine in a study.^[52]^ A total of 109 patients receiving hemodialysis (85) and peritoneal dialysis (24) received 2 30 μg doses of Pfizer mRNA vaccine (BNT162b2). The injections were administered intramuscularly 28 days apart, and patients who tested negative for anti-coronavirus antibodies after the second dose received the third dose 1 month later. Of the 102 patients followed up the following month, 91 had antibodies against COVID-19. The seropositive conversion rate of antibody-negative patients after the first dose was 88.7% (86 out of 97 patients). The acceptance of immunosuppressive therapy is an independent predictor of non-response to vaccination. Therefore, mRNA vaccines are believed to be highly immunogenic and safe. It is recommended that 2 doses of the vaccine be prioritized for dialysis patients. Those who do not respond to these 2 doses may require a third dose. Bertrand et al reported that 225 kidney transplant patients and 45 hemodialysis patients received 2 injections of the Pfizer mRNA vaccine (BNT162b2).^[53]^ The results suggest that hemodialysis patients have a higher response to the vaccine, whereas kidney transplant patients are affected by immunosuppressive agents (mainly tacrolimus and belatacept) and have a slightly poorer response to the vaccine. Among them, the specific T cell response in hemodialysis patients was 100% compared to 57.8% in kidney transplant patients. The specific effect of immunosuppressive agents on the immune response to vaccines is not yet clear, but for kidney transplant patients who have long-term use of immunosuppressants, the dose and complications of the vaccine need to be considered.

The above data show that the positivity rate of antibodies in patients with CKD after vaccination is lower than that in the general population, but there is still a positivity rate and a general tolerance to the vaccine; therefore, the effectiveness of vaccination should be considered. This can be achieved by choosing an appropriate and efficient vaccine or by increasing the vaccine dose.

Considering that the benefits of COVID-19 vaccination for patients with CKD outweigh the risks, it is recommended that patients with stable CKD should consider completing the vaccination plan, but doctors should be aware of the possibility of recurrence or deterioration of kidney disease and close monitoring.

### 3.6 Prospects for the application of the COVID-19 vaccine in patients with CKD.

Unified planning of vaccination is important for hemodialysis patients to avoid COVID-19 infection. Doctors, nurses, and pharmacists from multiple dialysis centers in Singapore formed volunteer groups to complete the procurement, logistics, and delivery of vaccines. During dialysis, the patients were vaccinated simultaneously. A total of 1500 cases of vaccinations were completed within 16 days, reducing the risk of infection at other vaccination centers.^[54]^ This indicates that hemodialysis patients could be given priority for a unified vaccination.

As mentioned earlier, patients with CKD, as a special vulnerable group, have a higher risk of COVID-19 infection, hospitalization, and mortality. However, at present, there are still certain restrictions on COVID-19 vaccination for patients with kidney disease in some countries, such as patients with severe kidney disease (uncontrolled acute and chronic nephritis, kidney disease treated with glucocorticoids, immunosuppressants, or biological agents; acute renal insufficiency or CKD stage 3 or above, that is, glomerular filtration rate (eGFR) <60 mL/min) are not recommended or allowed to receive the COVID-19 vaccine.^[55]^ This suggests that a large number of patients with CKD in some countries, especially those with ESRD, have not received the COVID-19 vaccine.

The British Nephrology Association's guidelines on the vaccination of patients with CKD indicate that the COVID-19 vaccination applies to all patients receiving hemodialysis, peritoneal dialysis, and kidney transplantation; CKD stage 5 patients who have not yet received dialysis; and kidney disease patients receiving immunosuppressive therapy. Immunosuppression treatment includes hormones (equivalent to prednisolone 20 mg/day, ≥4 weeks), lower doses of hormones combined with other immunosuppressive drugs, and receiving rituximab within the past 6 months.^[56]^ Thus, the indications and contraindications for COVID-19 vaccines for patients with CKD vary across regions. To protect patients, indications and contraindications should be further explored.

## 4 Conclusions

Patients with CKD are at high risk for COVID-19. Vaccination is a powerful weapon to protect CKD patients from COVID-19, but it may cause recurrence or deterioration of kidney disease. Therefore, patients with stable CKD should complete a vaccination plan, and doctors should pay attention to recurrence and deterioration of kidney disease and close monitoring.

## Acknowledgments

We thank the people who cooperated in performing this review.

## Author contributions

**Conceptualization:** Shuangxin Liu, Tang Bin.

**Data curation:** Bohou Li, Fengxia Zhang, Qiong Wu.

**Funding acquisition:** Shuangxin Liu, Wei Shi.

**Methodology:** Fengxia Zhang, Shuangxin Liu, Nan Jiang.

**Supervision:** Li Zuo, shuangxin liu, Wei Shi, Jiang Nan.

**Visualization:** Li Zuo.

**Writing – original draft:** Wanbing Huang, Tang Bin, Shuangxin liu

**Writing – review & editing:** Bohou Li, Nan Jiang, Tang Bin.
